# *MusaWRKY71* Overexpression in Banana Plants Leads to Altered Abiotic and Biotic Stress Responses

**DOI:** 10.1371/journal.pone.0075506

**Published:** 2013-10-08

**Authors:** Upendra K. S. Shekhawat, Thumballi R. Ganapathi

**Affiliations:** Plant Cell Culture Technology Section, Nuclear Agriculture and Biotechnology Division, Bhabha Atomic Research Centre, Trombay, Mumbai, India; National Taiwan University, Taiwan

## Abstract

WRKY transcription factors are specifically involved in the transcriptional reprogramming following incidence of abiotic or biotic stress on plants. We have previously documented a novel *WRKY* gene from banana, *MusaWRKY71*, which was inducible in response to a wide array of abiotic or biotic stress stimuli. The present work details the effects of *MusaWRKY71* overexpression in transgenic banana plants. Stable integration and overexpression of *MusaWRKY71* in transgenic banana plants was proved by Southern blot analysis and quantitative real time PCR. Transgenic banana plants overexpressing *MusaWRKY71* displayed enhanced tolerance towards oxidative and salt stress as indicated by better photosynthesis efficiency (Fv/Fm) and lower membrane damage of the assayed leaves. Further, differential regulation of putative downstream genes of MusaWRKY71 was investigated using real-time RT-PCR expression analysis. Out of a total of 122 genes belonging to *WRKY*, *pathogenesis-related (PR) protein genes*, *non-expressor of pathogenesis-related genes 1* (*NPR1*) and *chitinase* families analyzed, 10 genes (six belonging to *WRKY* family, three belonging to *PR proteins* family and one belonging to *chitinase* family) showed significant differential regulation in *MusaWRKY71* overexpressing lines. These results indicate that *MusaWRKY71* is an important constituent in the transcriptional reprogramming involved in diverse stress responses in banana.

## Introduction

Plant growth and development are adversely affected by different abiotic and biotic stress factors. In order to survive these stresses, plants have evolved the capacity to sense and react to these diverse external signals by means of specialized physiological and biochemical strategies. Upon stress perception, plants trigger a cascade of cellular events involving several parallel transduction pathways that eventually modulate the level of specific transcription factors resulting finally in the up- or down-regulation of genes coding for synthesis of effector proteins and/or metabolites which participate in stress tolerance [1]. Among these transcription factors, WRKY transcription factor family has been studied widely in several plant species [2]. WRKY transcription factors constitute one of the biggest families of transcription factors which are specific to plants and are involved in a multitude of physiological processes notably the abiotic and biotic stress responses [3]. We have previously conducted detailed studies on *MusaWRKY71* from banana focusing mainly on its inducibilty under different stress conditions. *MusaWRKY71* transcripts in banana plants were found to be up-regulated by cold, dehydration, salt, ABA, H_2_O_2_, ethylene, salicylic acid (SA) and methyl jasmonate (MJ) [4]. Stress inducible expression of this *WRKY* gene led us to postulate that a positive correlation may exist between *MusaWRKY71* expression and stress tolerance in banana. Further, several reports published recently indicate that overexpression of select specific *WRKY* genes in transgenic plants can lead to significantly improved stress tolerance in plants. *VvWRKY1* overexpression in grapes activated the expression of jasmonic acid pathway-related genes and improved the tolerance to the downy mildew [5]. Overexpression of *ZmWRKY33* in *Arabidopsis* induced *RD29A* known to be involved in stress-signaling and enhanced salt stress tolerance in the transgenic plants [6]. *GhWRKY15* transcript was mainly induced in cotton seedlings in response to biotic stress modulators like salicylic acid and methyl jasmonate and could impart tolerance to fungal pathogens in transgenic tobacco plants [7]. *TaWRKY2* and *TaWRKY19* of wheat imparted abiotic stress tolerance in transgenic *Arabidopsis* plants [8]. *CaWRKY40* overexpression in tobacco led to enhanced resistance to *Ralstonia solanacearum* and tolerance to heat shock whereas it’s silencing in pepper led to enhanced susceptibility to *R. solanacearum* and lowered thermotolerance [9].

Banana (*Musa spp.*) is one of the most important food and fruit crops in the world and is widely grown in many of the tropical countries. Although research in banana has been mostly concentrated on unraveling the ripening-related processes [10], several reports in the last few years have demonstrated the role of specific proteins in banana stress response pathways [11], [12]. In the present study, we have overexpressed *MusaWRKY71* in transgenic banana plants and shown its involvement in oxidative and salt stress tolerance. Further, differential expression of several putative *MusaWRKY71* target genes involved in biotic stress response pathways has been studied in detail.

## Results

### Generation of *MusaWRKY71* Overexpressing Banana Plants

**Figure 1:**
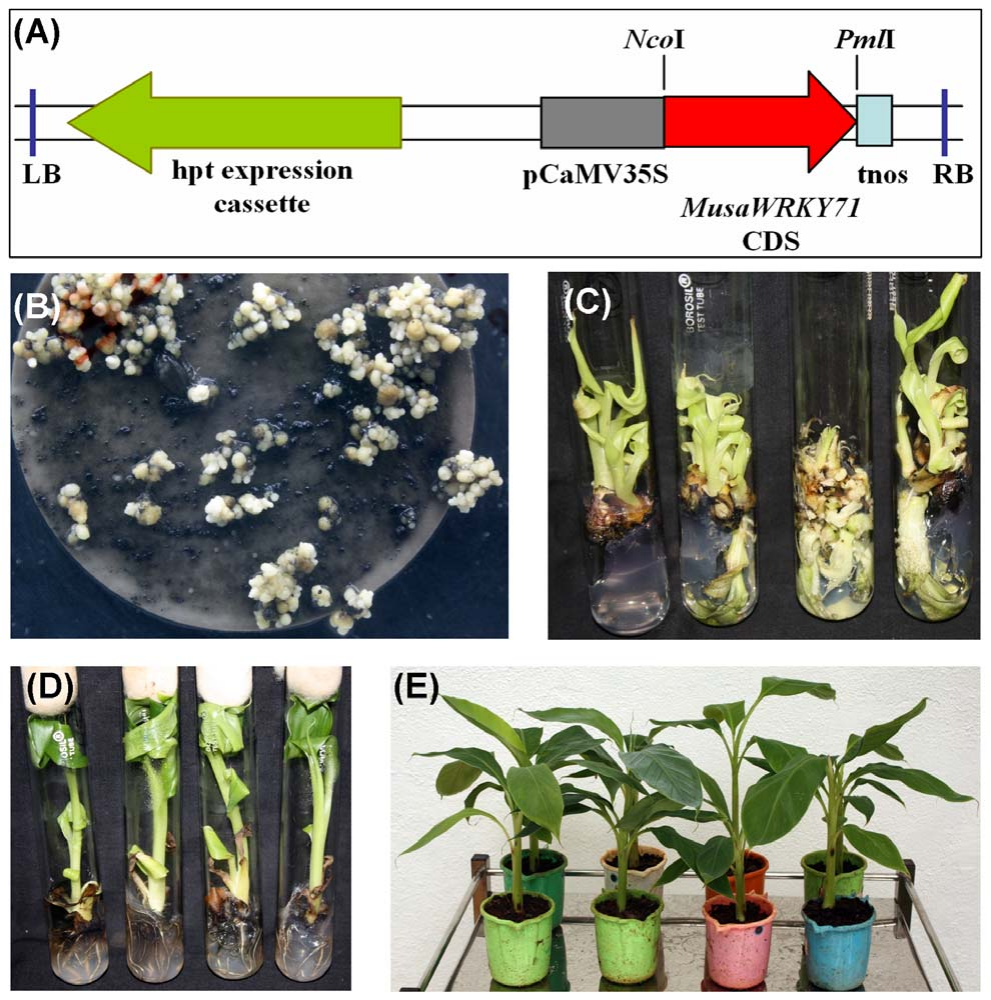
Generation of transgenic banana cv. *Rasthali* plants overexpressing *MusaWRKY71*. (A) T-DNA region of binary vector p1301-*MusaWRKY71* designed to constitutively overexpress *MusaWRKY71* in transgenic banana plants. (B) Transformed embryos on embryo induction medium. (C) Transgenic multiple shoots on multiple shoot induction medium. (D) Transgenic rooted plantlets on rooting medium. (E) Transgenic hardened plants in greenhouse (2-months old).

Banana cv. *Rasthali* embryogenic cells derived from 7 days old subcultured suspension cultures were cocultivated with *Agrobacterium* harboring p1301-*MusaWRKY71* plant expression vector [4] designed to overexpress *MusaWRKY71* in a constitutive manner in the transgenic banana plants (Figure 1A). Three to four weeks after cocultivation, whitish translucent embryos developed on banana embryo induction medium supplemented with hygromycin (5 mg l^−1^). Secondary embryos also developed from these primary embryos upon subculturing onto fresh medium of the same composition (Figure 1B). These embryos were subcultured onto embryo germination medium containing BAP for efficient germination. The germinating embryos were then transferred on to banana multiplication medium to facilitate multiple shoot induction (Figure 1C). The clonal shoots developed for each transgenic line were separated and rooted on MS medium supplemented with NAA (Figure 1D). Rooted plantlets were acclimatized in a contained greenhouse (Figure 1E).

### Molecular Analysis of the Transgenic Plants

**Figure 2:**
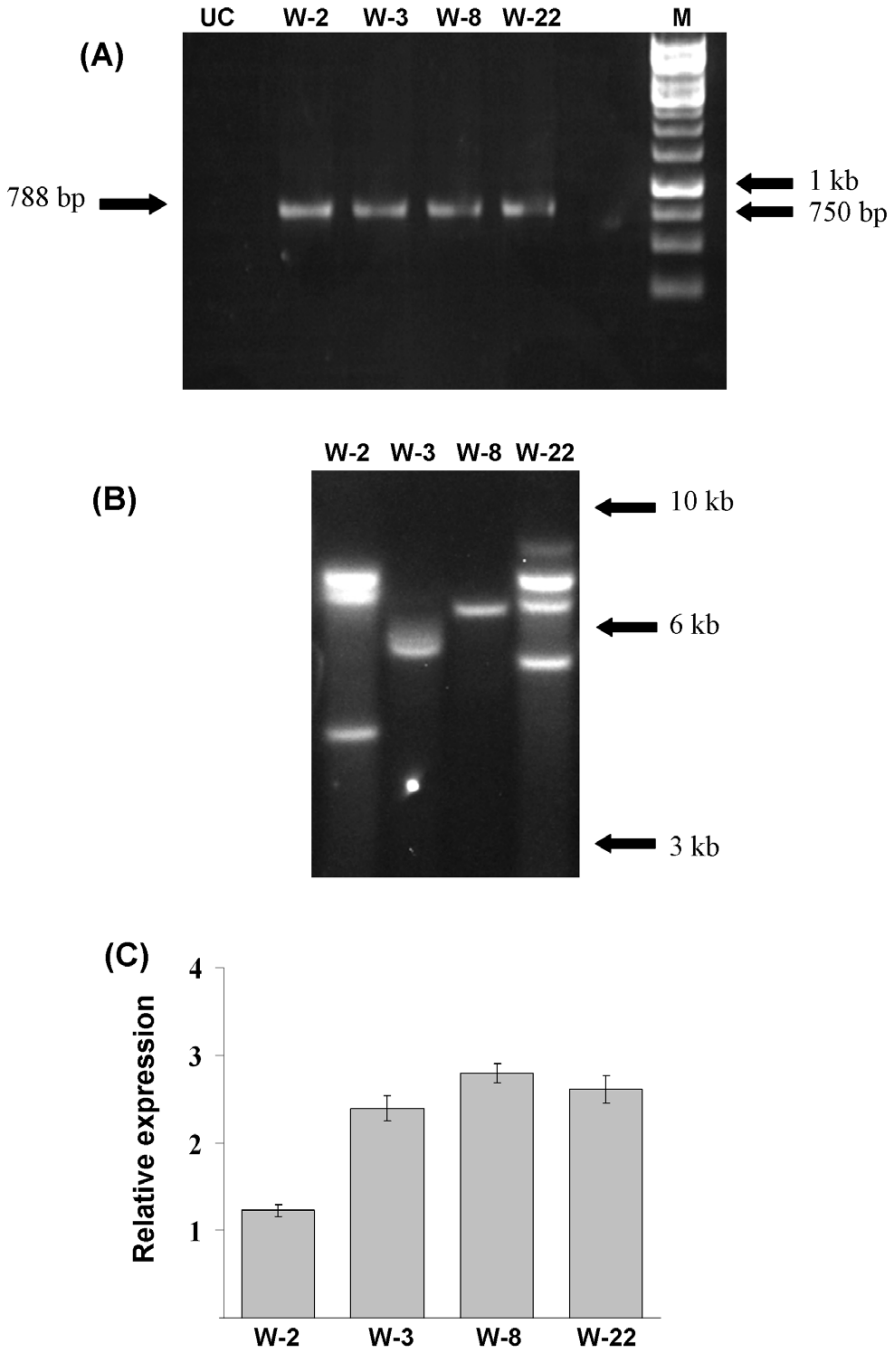
Molecular analysis of putatively transgenic banana plants. (A) Genomic DNA*-*PCR analysis of untransformed control (UC) and the transgenic lines (W2, W3, W8 and W22). (B) Southern blot analysis of p1301-*MusaWRKY71* transformed banana lines. (C) Real-time quantitative RT-PCR analysis of the selected transgenic lines (W2, W3, W8 and W22) for determination of the exact quantum of *MusaWRKY71* overexpression in transgenic banana lines. All gene expression values have been normalized against *Musa EF1α* cDNA expression levels. Expression of *MusaWRKY71* in untransformed plants has been assumed to be 1 for estimating the level of overexpression of *MusaWRKY71* in different transgenic lines. Values are mean ± SE.

Seven transgenic lines were observed to grow successfully upon repeated subcultures in hygromycin containing medium. Genomic DNA PCR analysis of these putative *MusaWRKY71* overexpressing lines showed a single 788-bp amplified fragment derived from hygromycin phosphotransferase coding sequence whereas it was absent in untransformed control plants (Figure 2A). Four randomly selected lines (W2, W3, W8 and W22) were characterized by Southern blotting wherein restriction digested genomic DNA was probed with a DIG-labeled probe generated using hygromycin phosphotransferase gene coding region. Restriction enzyme *BamH*I was used to restrict the genomic DNAs as it is expected to cut the T-DNA region of the overexpression vector (p1301-*MusaWRKY71*) only once and therefore the number of chemiluminescent bands obtained can directly be taken as the number of T-DNA copies transferred to banana genome in these transgenic lines. T-DNA copy numbers in the range of 1 to 4 were found in these four lines (Figure 2B). Quantitative real-time RT-PCR was employed to estimate the exact quantum of overexpression of *MusaWRKY71* transcript in the four selected lines. *MusaWRKY71* overexpression, determined by using *Musa EF1α* gene for expression normalization, was found to be 1.231 times (relative to the expression of *MusaWRKY71* in untranformed control) in W2, 2.395 times in W3, 2.789 times in W8 and 2.610 times in W22 line (Figure 2C).

### Detached Leaf Stress Tolerance Assays for *MusaWRKY71* Overexpressing Lines

**Figure 3:**
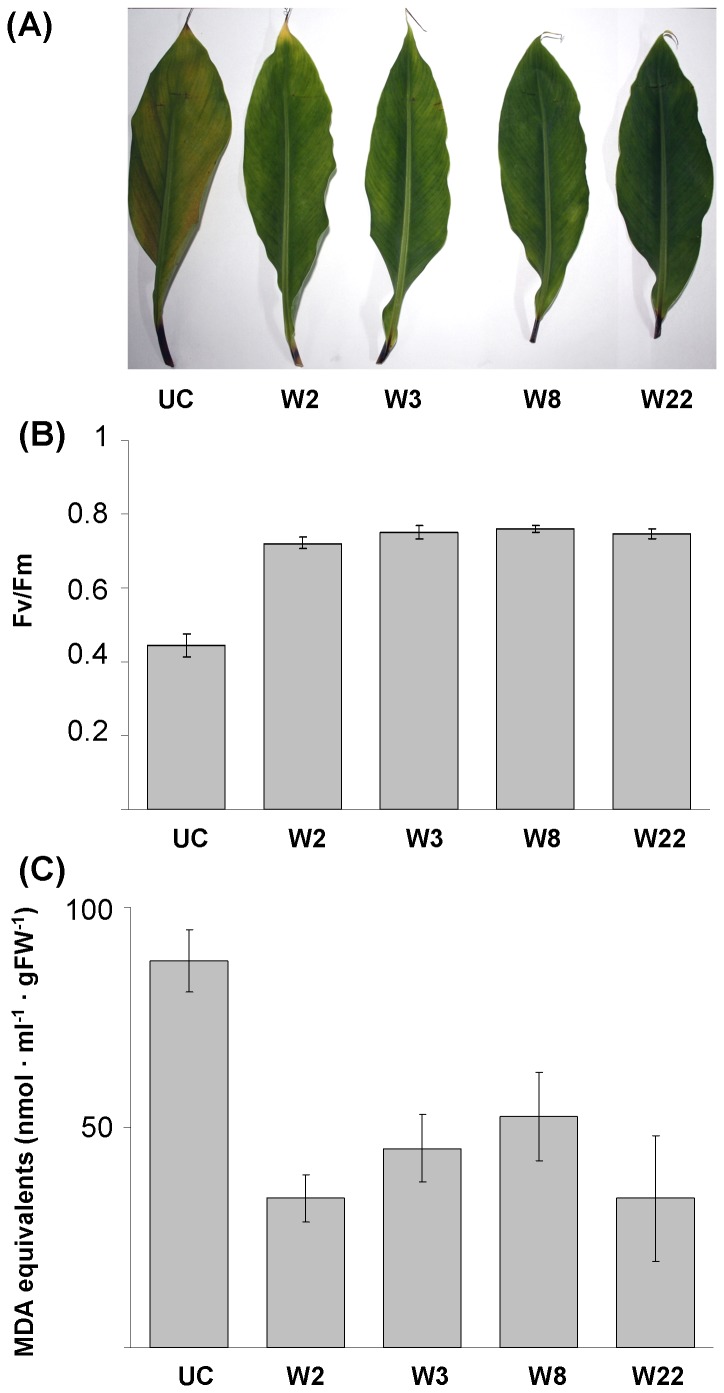
Detached leaf oxidative stress assay of p1301-*MusaWRKY71* transgenic plants. (A) Detached banana leaves derived from greenhouse maintained transgenic (W2, W3, W8 and W22) and control plants (UC) after exposure to simulated oxidative stress (10 µM methyl viologen in 1/10 MS basal medium for 7 days). (B) Photosynthetic efficiency (measured as Fv/Fm ratio) of untransformed and p1301-*MusaWRKY71* transgenic leaves exposed to methyl viologen. (C) MDA levels in untransformed and p1301-*MusaWRKY71* transgenic leaves exposed to methyl viologen.

**Figure 4:**
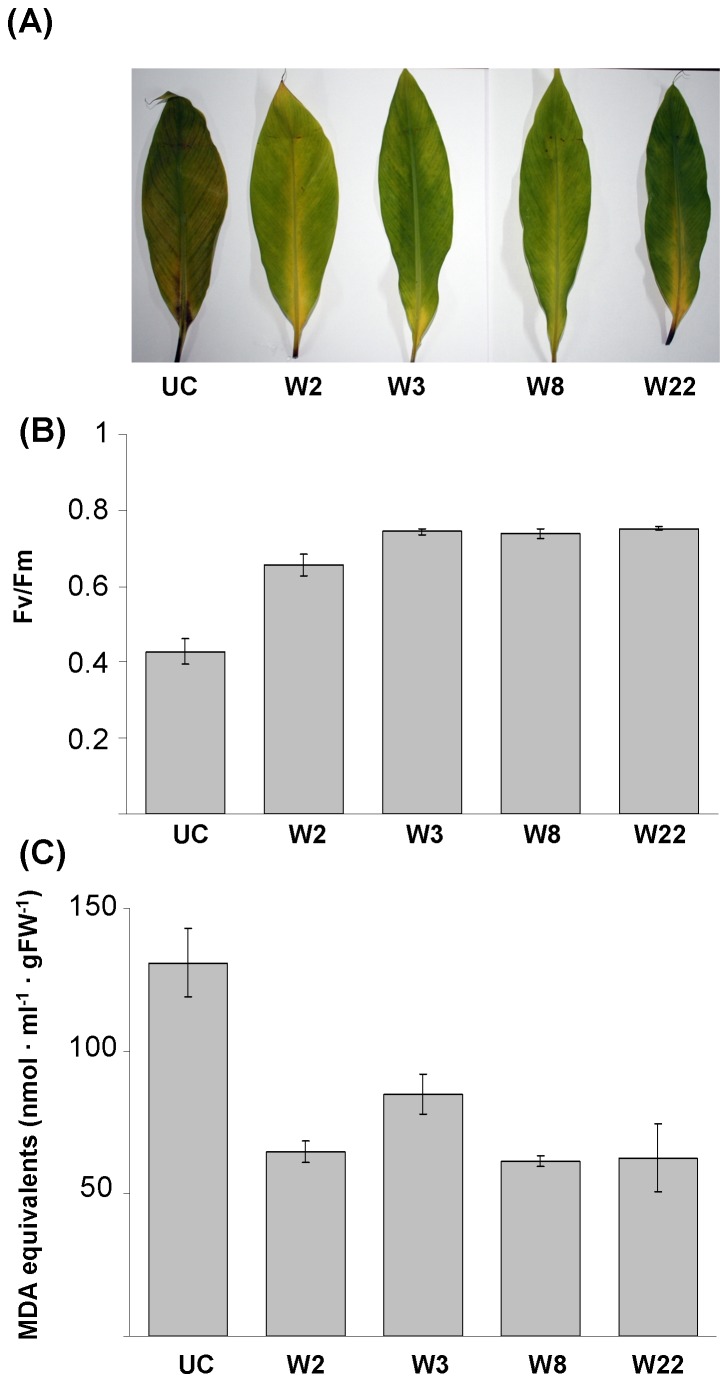
Detached leaf salt stress assay of p1301-*MusaWRKY71* transgenic plants. (A) Detached banana leaves derived from greenhouse maintained transgenic (W2, W3, W8 and W22) and control plants (UC) after exposure to simulated salt stress (350 mM NaCl in 1/10 MS basal medium for 7 days). (B) Photosynthetic efficiency (measured as Fv/Fm ratio) of untransformed and p1301-*MusaWRKY71* transgenic leaves exposed to salt. (C) MDA levels in untransformed and p1301-*MusaWRKY71* transgenic leaves exposed to salt.

Transgenic banana plants overexpressing *MusaWRKY71* were observed to be phenotypically normal and indistinguishable from the untransformed plants in both *in vitro* and *ex vitro* conditions. To assess the stress hardiness of *MusaWRKY71* overexpressing plants, we used detached leaves from greenhouse grown transgenic and untransformed control plants in stress assays. Detached leaves along with their petioles were exposed to 10 µM methyl viologen or 350 mM NaCl in 1/10 MS medium for 7 days. Leaves derived from *MusaWRKY71* overexpressing transgenic plants showed significantly less damage by the simulated oxidative or salt stress treatments as compared to untransformed control leaves which showed marked browning and chlorosis respectively in oxidative and salt stress application (Figure 3A, Figure 4A). Oxidative stress exerted through ROS is a common constituent of most abiotic as well as biotic stress conditions. Methyl viologen accepts electrons from photosystem I in the presence of light and transfers the same to oxygen to generate ROS. Treatment with methyl viologen and NaCl is a standard procedure employed to examine improved tolerance against oxidative and salt stress in plants and the fact that detached transgenic leaves remained green and looked better than equivalent controls even after 7 days in methyl viologen or NaCl solution clearly proved that *MusaWRKY71* overexpression in these transgenic plants resulted in an improved ability to tolerate these stress conditions. Photosynthetic efficiency quantified in the form of maximum quantum efficiency of Photosystem II and denoted as physiological parameter Fv/Fm [ratio of variable fluorescence (Fv) over the maximum fluorescence value (Fm)] is a sensitive marker of overall plant wellbeing and exposure to any form of abiotic or biotic stress stimuli causes reduction in photochemical quenching of energy within Photosystem II leading to a lowered Fv/Fm. The stressed leaves derived from untransformed control plants displayed the lowest Fv/Fm in both the stress assays suggesting deficient photosynthetic functions in these leaves as compared to the *MusaWRKY71* overexpressing transgenic leaves (Figure 3B, Figure 4B). The damage caused to the cellular membranes (including chloroplastic membranes) during detached leaf assays was also quantified by TBARS (Thiobarbituric Acid Reactive Substances) assay which estimates the level of malondialdehyde (MDA) formed by lipid peroxidation of cellular membranes. Untransformed control leaves showed the maximum damage to cellular membranes as the MDA levels were found to be the highest in these leaves (Figure 3C, Figure 4C). Constitutive overexpression of *MusaWRKY71* in transgenic plants thus enhanced the capacity to scavenge the reactive oxygen species, thereby minimizing the damage inflicted by these free radicals on the target cellular membranes of transgenic banana plants.

### Differential Expression of Putative MusaWRKY71 Target Genes in *MusaWRKY71* Overexpressing Lines

**Figure 5:**
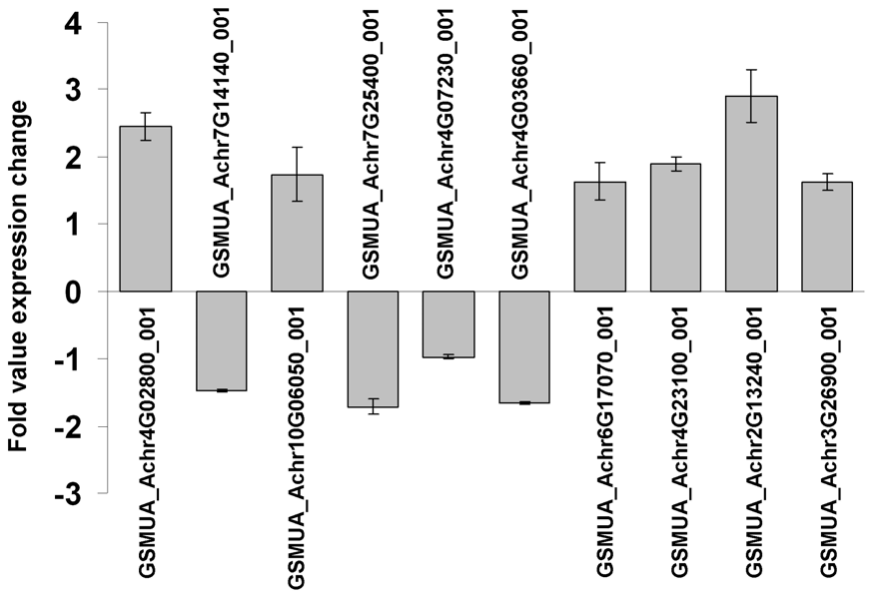
Differential regulation of putative MusaWRKY71 downstream genes in *MusaWRKY71* overexpressing plants. Six *WRKY* genes (GSMUA_Achr4G02800_001, GSMUA_Achr7G14140_001, GSMUA_Achr10G06050_001, GSMUA_Achr7G25400_001, GSMUA_Achr4G07230_001, GSMUA_Achr4G03660_001), 3 *PR protein* genes (GSMUA_Achr6G17070_001, GSMUA_Achr4G23100_001, GSMUA_Achr2G13240_001), and 1 *chitinase* gene (GSMUA_Achr3G26900_001) showed differential expression in the transgenic plants. All gene expression values have been normalized against *Musa EF1α* cDNA expression levels. The x-axis represents the expression level of *MusaWRKY71* in control conditions. Values are mean ± SE.

Although *MusaWRKY71* is induced in response to elicitor molecules of biotic stress response pathways like ethylene, SA and MJ, the *MusaWRKY71* overexpressing plants were found to be equally susceptible to the infection of *Fusarium oxysporum* f. sp. *cubense* as the untransformed control plants (Figure S1). Thus, to gain clues for understanding the role of *MusaWRKY71* in biotic stress responses of banana plant, we attempted to identify those defense related genes whose expression is modulated by *MusaWRKY71* overexpression. Among the most probable downstream targets of WRKY proteins are other *WRKY* genes (*WRKY* genes are known to be cross regulated by other members of the wider *WRKY* gene family in a plant species), *PR protein* genes, *NPR1* genes and *chitinase* genes. We studied the differential regulation of these gene families in *MusaWRKY71* overexpressing plants by using specific primers in quantitative RT-PCR (Figure 5). Among the 67 *WRKY* genes analyzed, differential expression was observed in six genes. Whereas the expression of GSMUA_Achr4G02800_001 and GSMUA_Achr10G06050_001 was upregulated respectively by 2.45 and 1.74 folds, the expression of GSMUA_Achr7G14140_001 GSMUA_Achr7G25400_001, GSMUA_Achr4G07230_001 and GSMUA_Achr4G03660_001 was down regulated respectively by 1.47, 1.71, 0.97 and 1.67 folds. Similarly, among the 20 *PR protein* genes tested, three genes were found to be up regulated following *MusaWRKY71* overexpression. The expression of GSMUA_Achr6G17070_001, GSMUA_Achr4G23100_001 and GSMUA_Achr2G13240_001 was respectively up regulated by 1.64, 1.9 and 2.9 folds. Further, among the 26 chitinase genes tested the expression of only GSMUA_Achr3G26900_001 was found to be upregulated by 1.6 fold in *MusaWRKY71* overexpressing plants. No difference in expression of *NPR1* genes was noticed between the *MusaWRKY71* overexpressing and untransformed control plants (data not shown). Further, all the genes wherein differential expression was noticed had WRKY protein binding W-box like sequences [(C/T)TGAC(T/C)] in the 1 kb proximal promoter regions.

## Discussion

Transcriptional modulation of various plant effector genes is crucial for activation of plant inducible stress responses. Hence, understanding the intricate regulation of plant stress response mechanisms by identification of different regulatory components and their respective roles in transcriptional regulatory pathways assumes significance. Research in model plant systems like *Arabidopsis* and rice has established that members of several families of transcription factors like ethylene-responsive element binding proteins, myb-like proteins, bZIP proteins, and WRKY proteins are likely involved in the transcriptional regulation of plant stress response genes [13]. Among these, members of *WRKY* gene family have been shown to be involved in both biotic and abiotic stress responses of plants [14], [15]. Since the first WRKY protein (SPF1) was identified in sweet potato [16], the functions of several WRKY transcriptional factors have been elucidated in the context of plant stress responses [3]. Most of these have been derived from dicot model plants like *Arabidopsis*, tomato, tobacco and potato whereas monocots as a group have lagged behind despite their economic importance. Valuable fruit crops like banana were not studied in this perspective and hence, investigation into functions of WRKY proteins in banana stress response and signaling assumes importance as banana is the most important crop in the world and because of vulnerability to biotic and abiotic stresses.

In an earlier study, we had identified a banana EST sequence corresponding to a *WRKY* gene based on comparative analysis of stressed and non-stressed tissue derived EST data sets [4]. This *WRKY* gene (*MusaWRKY71*) was further extended towards the 3′ end to get the complete gene sequence. MusaWRKY71 was observed to be nuclear localized and its transcription was inducible in response to a variety of abiotic and biotic stress conditions. To further characterize its functions in banana stress response pathways, we overexpressed this gene in transgenic banana plants as the best method to examine the functions of a protein is its overexpression in the parent plant species wherein direct study of its interactions with other macromolecules present in the native plant is possible. The *MusaWRKY71* overexpressing transgenic banana grew normally in vitro in the presence of hygromycin selection and no gross abnormalities or stunting effects were noticed. Leaves derived from these transgenic plants performed better in the abiotic stress assays as demonstrated by lower phenotypic damage and positive membrane damage and photosynthetic efficiency parameters as compared to controls. When these plants were subjected to Fusarium wilt assay, we did not observe any difference between the untransformed controls and the four transgenic lines indicating that overexpression of *MusaWRKY71* was not sufficient to modulate the plant defenses to achieve Fusarium tolerance. Failure to observe any difference with respect to biotic stress exerted by Fusarium, and our previous observation regarding the SA, MJ and ethephon inducibility of *MusaWRKY71* led us to look for differential expression of genes which are known to be involved in biotic stress response pathways. Among the genes which are known downstream targets of WRKY proteins, genes of *WRKY* [17], *PR proteins* [18], *chitinases* [19] and *NPR1 proteins* [20] have been studied in detail in relation to biotic stress response pathways. Our results showing differential expression of a total of 10 genes out of a total of 122 tested from the above four groups indicated that although *MusaWRKY71* by itself is unable to prevent a fatal biotic interaction, but it still controls the expression of other genes related to biotic stress. Together with the fact that *MusaWRKY71* overexpressing plants were tolerant towards oxidative and salt stress, the differential expression of several biotic stress related genes pointed towards a role for this protein in the interface between the abiotic and biotic stress response pathways of banana plant.

Over the last decade several studies detailing overexpression and downregulation of select specific *WRKY* genes have been conducted in different plant species. *OsWRKY72* overexpressing plants were retarded and displayed early flowering as well as reduced apical dominance along with an enhanced gravitropism response [21]. Further the plants were more sensitive towards salt and osmotic stress. *Arabidopsis* ABO3, a WRKY transcription factor was found to mediate plant responses to abscisic acid and drought tolerance, as an ABA overly sensitive mutant (*abo3*) was hypersensitive to ABA in seedling establishment and growth [22]. *CaWRKY1*- overexpressing transgenic plants showed heightened hypersensitive cell death in response to tobacco mosaic virus and *Pseudomonas syringe* pv. *tabaci* [23]. *CaWRKY1* was in fact observed to act as a regulator to turn off systemic acquired resistance once the pathogen challenge has diminished. Overexpression of *OsWRKY89* in transgenic rice increased lignification and reduced internode length [24]. It also enhanced resistance to the rice blast fungus and the tolerance to UV-B irradiation. Constitutive overexpression of *VvWRKY2* in transgenic tobacco plants reduced the susceptibility towards different fungal pathogens indicating the role for *WRKY* genes in fungal tolerance in plants [25]. The varied phenotypes and physiological effects resulting from modulation of *WRKY* expression in transgenic plants showed the multiple roles of these proteins in plants. Further, in our study at least one gene from the three out of four groups of genes (*WRKY*, *PR proteins* and *Chitinases*) was found to be differentially regulated. But among the nine *NPR1* genes tested, none of the genes were found to have differential expression in the transgenic lines. In contrast, the rice plants overexpressing *OsWRKY71* (the closest homologue of *MusaWRKY71*) showed enhanced expression of *OsNPR1* and *OsPR1b* as well as improved resistance towards rice bacterial blight [26]. Thus, it appears that even among related monocot species like rice and banana, the regulation of defense signaling pathways involving *WRKY* and *NPR1* genes can be quite divergent.

In conclusion, *MusaWRKY71* is proposed to be an important member of the *WRKY* gene family in banana which has the capacity to cross regulate other *WRKY* gene members as well as other genes involved in biotic stress signaling pathways. Further, since overexpression oof this gene provides the transgenic plants with an improved oxidative and salt stress tolerance, it may well be a significant link in the interface between abiotic and biotic stress pathways. Further studies involving microarray experiments based on the newly uncovered banana genome sequence [27] are warranted in future to fully establish the multi faceted roles played by *MusaWRKY71* gene in banana stress responses.

## Materials and Methods

### Generation of *MusaWRKY71* Overexpressing Banana Plants

p1301-*MusaWRKY71* binary vector described earlier [4] was used to transform banana cv. *Rasthali* embryogeneic cell suspension cultures as essentially described earlier [28]. The binary vector was transferred to *Agrobacterium tumefaciens* EHA105 by electroporation and this *Agrobacterium* culture was then cocultivated with 0.5 ml packed cell volume of banana embryogenic cells. These cells were then aspirated onto glass fibre filters and cultured on to solidified M2 medium added with 100 µM ACS. After three days of incubation in dark at 25±1°C, the cells (along with the filters) were transferred to fresh M2 medium added with cefotaxime (400 mg l^−1^). Three days later the cells were removed from the filters and transferred onto banana embryo induction medium supplemented with cefotaxime (400 mg l^−1^) and hygromycin (5 mg l^−1^). Somatic embryos developed in 3 to 4 weeks were then cultured on the same medium for three subculture routines of three weeks duration on hygromycin selection medium. Developed embryos were then transferred to MS medium supplemented with BAP (0.5 mg l^−1^) for germination. Rapidly developing embryos were subsequently transferred to banana multiplication medium to multiply each putatively transformed line. Individual shoots isolated from the multiple shoot cultures were transferred to MS medium added with NAA (1 mg l^−1^) for rooting. These tissue culture generated plantlets were then hardened in the greenhouse and used for all the analysis.

### Molecular Analysis of the Transgenic Plants

Genomic DNA isolated from seven putatively transformed banana lines actively growing on hygromycin containing medium using GenElute Genomic DNA miniprep kit (Sigma, USA) was used as template in PCR with primers specific for hygromycin phosphotransferase gene present within the T-DNA borders of p1301-*MusaWRKY71* binary vector. Genomic DNA isolated from untransformed banana plant served as negative control in these PCR. Four lines were then randomly chosen from the lines which showed bands specific for hygromycin phosphotransferase gene. These four lines (W2, W3, W8 and W22) were then analyzed by southern blot analysis (performed using DIG-labeled probes specific for hygromycin phosphotransferase gene) for estimation of T-DNA copy numbers in these lines. Chemiluminescence was detected on the nylon filters using a chemiluminescence enabled gel-documentation system. The exact quantum of overexpression of *MusaWRKY71* in these four selected transgenic lines was examined by real time quantitative RT-PCR analysis. Total RNA extraction from young leaves of transgenic plants and first strand cDNA synthesis were done as described before [11]. Samples derived from three independent plants of each line were pooled prior to RNA isolation to ensure reproducibility of results. cDNA derived from untransformed banana plants were used as controls in these RT-PCR performed using SYBR Green Extract-N-Amp PCR ReadyMix (Sigma, USA). *Musa acuminata* EF 1α gene [29] was amplified together with *MusaWRKY71* gene to allow gene-expression normalization and subsequent quantification. All real-time quantitative RT-PCR were performed using Qiagen make Rotor-Gene Q platform. PCR Cycling parameters for real time RT-PCR were 94°C for 10 min initially followed by 40 PCR cycles, each comprising of 94°C for 30 seconds, 56°C for 30 seconds and 72°C for 30 seconds. Ct values obtained from Rotor-Gene Q software were analyzed using REST-MCS software [30] to arrive at relative expression level values for MusaWRKY71 gene in different overexpression lines.

### Detached Leaf Stress Tolerance Assays for *MusaWRKY71* Overexpressing Lines

Deatched leaves derived from 2–3 months old transgenic hardened *MusaWRKY71* overexpressing lines were used in assays for improved drought and salt tolerance. Leaves were excised along with their petioles and their cut ends were dipped in 1/10 MS medium added individually with methyl viologen (10µM) or NaCl (350 mM) for 7 days in 16 hour light/8 hour dark regime. Detached leaves so treated with methyl viologen or NaCl were then analysed for membrane damage by estimating their malondialdehyde (MDA) content [11]. Further, continuous excitation plant efficiency analyzer (Hansatech Instruments make Model no. Handy-Pea) was used to estimate photosynthetic efficiency (Fv/Fm) in all detached leaf samples. Leaves derived from hardened greenhouse maintained plants of untransformed banana cv. *Rasthali* were used as controls in all the above assays. All the stress assays were conducted in triplicates and after their completion representative samples for each assay were photographed.

### Differential Expression of Putative *MusaWRKY71* Target Genes in *MusaWRKY71* Overexpressing Lines

To investigate the effect of *MusaWRKY71* on expression of other *WRKY* gene family members in banana, cDNA isolated from leaves of *MusaWRKY71* overexpressing banana lines was utilized in real time quantitative RT-PCR together with primers specific for 67 annotated *WRKY* genes (Table S1) from banana genome project [27]. cDNA isolated from untransformed control plants was used as control in these reactions. Rest-MCS software was used to arrive at relative expression values [29] and primers specific to *EF1α* gene were used for normalization of expression values. Similarly, primers specific to 20 *PR protein* genes (Table S2), 26 *chitinase* genes (Table S3) and 9 *NPR1* genes (Table S4) were tested to determine their differential expression in *MusaWRKY71* overexpressing lines.

## Supporting Information

Figure S1 **Fusarium wilt reistance assay for** ***MusaWRKY71*** **overexpressing transgenic banana plants.** *Fusarium oxysporum* f. sp. *cubense* culture grown with sand-maize bran mix was used for infecting transgenic and untransformed control banana plants. Roots of these plants were injured before they were transferred to the sand-maize bran mix together with soil. Both the untransformed control plants and the transgenic plants showed typical wilt symptoms within one month. CON-Untransformed control plant; W2, W3, W8, W22– *MusaWRKY71* overexpressing transgenic banana lines.
(TIF)

Table S1 **WRKY genes and primers used in this study.**
(DOC)

Table S2 **PR protein genes and primers used in this study.**
(DOC)

Table S3 **Chitinase genes and primers used in this study.**
(DOC)

Table S4 **NPR1 genes and primers used in this study.**
(DOC)
