# Supplementary figures and images for: MusaWRKY71 Overexpression in Banana Plants Leads to Altered Abiotic and Biotic Stress Responses
Source: PLoS One. 2013 Oct 8;8(10):e75506. doi: 10.1371/journal.pone.0075506 (PMC3792942; doi:10.1371/journal.pone.0075506)

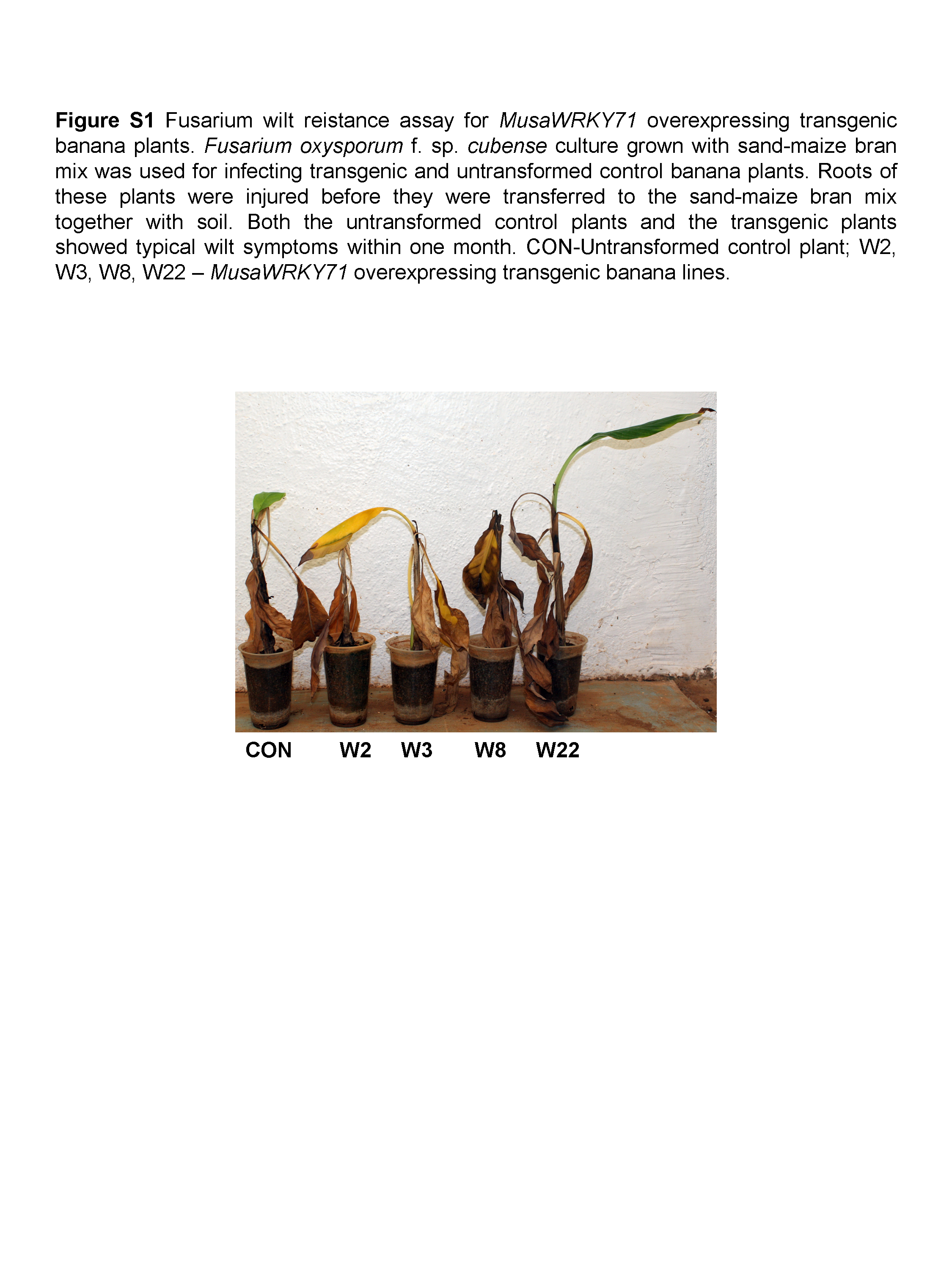

Supplement: Figure S1 — Fusarium wilt reistance assay for MusaWRKY71 overexpressing transgenic banana plants. Fusarium oxysporum f. sp. cubense culture grown with sand-maize bran mix was used for infecting transgenic and untransformed control banana plants. Roots of these plants were injured before they were transferred to the sand-maize bran mix together with soil. Both the untransformed control plants and the transgenic plants showed typical wilt symptoms within one month. CON-Untransformed control plant; W2, W3, W8, W22– MusaWRKY71 overexpressing transgenic banana lines. (TIF) [file pone.0075506.s001.tif]
