# Supplementary material for: MusaWRKY71 Overexpression in Banana Plants Leads to Altered Abiotic and Biotic Stress Responses
Source: PLoS One. 2013 Oct 8;8(10):e75506. doi: 10.1371/journal.pone.0075506 (PMC3792942; doi:10.1371/journal.pone.0075506)
Supplement: Table S1 — WRKY genes and primers used in this study. (DOC) [file pone.0075506.s002.doc]

**Table S1** Banana WRKY genes and primers

| GSMUA_Achr3G00510_001 | AGCTTGGAGCACCTCGTCT |
| --- | --- |
|  | GGAAGGAGGAGTTCGTGGAC |
| GSMUA_Achr3G13440_001 | GATCGGCTTGGTTGGATCT |
|  | GAGGTGTACTTCGCCGTGAT |
| GSMUA_Achr1G27980_001 | CTGCTGCTGAGCTTTCGAG |
|  | AGCTCGCTGAATTGCTTCTG |
| GSMUA_Achr10G01150_001 | GCTGATGACGACTGGGATCT |
|  | ACTCCACCTCGACTGTTGCT |
| GSMUA_Achr3G29310_001 | GGAAGGAGTGGAAGACGCTAA |
|  | AGAAGATGCTGTGGTTGAAGC |
| GSMUA_Achr3G09940_001 | AGCCTCTGTCCACCAGAAAG |
|  | GGAGTCTGCACCACCACTCT |
| GSMUA_Achr5G16460_001 | ATTTAGAAGCCGAGCTGAACC |
|  | TCGTGTTCTTCGGGTCGAT |
| GSMUA_Achr1G17160_001 | CCTCGTCAGGTTCACAGGTC |
|  | GGTCGTCGTTCTTCCTTCTCT |
| GSMUA_Achr11G08290_001 | CCAATCCTCTCGTCCTGAAG |
|  | TTGGAGGAGGAGATGTCTCAG |
| GSMUA_Achr4G01600_001 | CAAACCTTGCCATAGCTGGA |
|  | AGCTGAGGTCCTAGAGGAAGC |
| GSMUA_Achr1G04770_001 | TCTCCTTCTACGCCGAGAGA |
|  | ACTGAAGAGCTCGGTGATGC |
| GSMUA_Achr6G22160_001 | TCATCAAAGCCCCGCAACT |
|  | CTCGCTGTACTGCGTGAAGG |
| GSMUA_Achr5G16750_001 | CAACTTCCTAGCGAGGACCA |
|  | GATTGGGTTTGGAGCGTCT |
| GSMUA_Achr4G14270_001 | TCAACGTCCGACACCAAATA |
|  | TCAACCTCGCTTGTTGTCTG |
| GSMUA_Achr10G22020_001 | AGTTACCCTCCCCTGAAGGA |
|  | CCACCAAGAGCAAGTCGTC |
| GSMUA_Achr9G22950_001 | GGAGGATGGACGACCAGAT |
|  | CTCTACCGGGGGTACGAAG |
| GSMUA_Achr10G24080_001 | AAGAGAACAGGAGGCTTACCG |
|  | CGGTTCGCACATAAACCTTC |
| GSMUA_Achr5G09430_001 | TCCTCCTCGAGCCCACTA |
|  | GTCGAAGTATCCGGAGCTGA |
| GSMUA_Achr4G16840_001 | GTGGATGAGCAAAACGAACC |
|  | ACCATCTTCCGTAACGCTGT |
| GSMUA_Achr10G11580_001 | GCGATCCAAGGAAGATGGTA |
|  | TGATGTTGTTTGTGAAGAAACTGA |
| GSMUA_Achr4G13970_001 | GCAGCAGCCTTTAGAAATCG |
|  | ACCAAATTTCCTGCAGTCGT |
| GSMUA_Achr10G15290_001 | CACGTTGGATCTCACCCAAA |
|  | AAGTTCGGGTCGGCAGTTAT |
| GSMUA_Achr4G20600_001 | TGACGGTTTCCAAGTTCAGG |
|  | CCGAGACTCAGGGTCGTTG |
| GSMUA_Achr6G31840_001 | CAGCACTCGCCAGAAGTTT |
|  | ATGTCTCCCACCGTCAACA |
| GSMUA_Achr6G16760_001 | AAATGGCATGACTGCTGCTAC |
|  | ACCTCGCTTGTTGTTTGCAC |
| GSMUA_Achr5G04150_001 | AGGCTTGACCAACCTTGCTA |
|  | CTAGAGGTAGCCTGCTCATCATC |
| GSMUA_Achr7G00610_001 | AGCGGGTCTAATGGTTCAAA |
|  | GATGCTGATTGGTTGTTTCTGA |
| GSMUA_AchrUn_randomG17930_001 | CCAAGTGCAACGTGAAGAAG |
|  | TTTTCGAGAGTAGGGACGATG |
| GSMUA_Achr7G26450_001 | AATCCTAACCTAGGCTCTCTGGA |
|  | TCTGAGAAGAAGCTGCCATGT |
| GSMUA_Achr3G15690_001 | CAGAACCGCCTACCTCCTC |
|  | CCGAGGTAGTGGAAGCAGAA |
| GSMUA_Achr7G15930_001 | GTTCATGTCGTCCGTCACC |
|  | TCACCCGTGATTTCCTTCTC |
| GSMUA_Achr10G08720_001 | CACTCCAACTCCTCCCAAGA |
|  | AGCTGGCAACACTCCTATCC |
| GSMUA_Achr8G20660_001 | GCTGGCCATGGAAGAGTTAG |
|  | GATCAGATGATGCTGGTCCAC |
| GSMUA_Achr3G29850_001 | ACCTTGGTGTTGGCATCAG |
|  | GCGGCCGAATAGTACATATCA |
| GSMUA_Achr11G17620_001 | TGACGCAGTCCGTGAACC |
|  | GGACTTGGGAAGCTGAGGAG |
| GSMUA_Achr6G02330_001 | AGCAGAAGCAGCAGCAAATC |
|  | TCCAAAGTTGCTCCAGTGTG |
| GSMUA_Achr5G21910_001 | CTTCGCTCAATGGAGCACCT |
|  | CGCGAGGGTAAGACTCTTCG |
| GSMUA_Achr1G23560_001 | CAACCTGGACTTCACCAACC |
|  | ATCATCGAGGGATCGCTTCT |
| GSMUA_Achr9G21560_001 | CCTTCACTTCCTCCAACAGC |
|  | TTCATCGAGTCGGACAACTG |
| GSMUA_Achr7G24840_001 | GAACCACCACCACAAGCAC |
|  | GCGAACAAAGAGTCCTCCTC |
| GSMUA_Achr6G27090_001 | GTAGTCGCAGGAGCAAGACC |
|  | TCCCTCTCCCTCTTCTCTCC |
| GSMUA_Achr4G02800_001 | CACACACCTAGTGGCACCTC |
|  | TGCCGTGATCAAAAGAGAATAGT |
| GSMUA_Achr9G06750_001 | GTGGTGATGGCGGTAAGAAC |
|  | CCTTTCTTCCTCGGCTTGTT |
| GSMUA_Achr7G14140_001 | CCTTCTCCTTCCCTTCGACT |
|  | AATGATCTCGGTGAGGTCAGA |
| GSMUA_Achr4G14990_001 | CAACAGTCAGAGGGAGCACA |
|  | GCCGTGAATGAAGGAAGGA |
| GSMUA_Achr3G27880_001 | GGTGTCGTCGGCCCATAAG |
|  | TTTTTCCCGGTCCTTCCTGT |
| GSMUA_Achr10G06050_001 | TGGCTGATCTGATATCCAAGG |
|  | GCTTCGAAACTTTGGGCTTA |
| GSMUA_Achr5G04870_001 | TTGACCCTCGACTTCACGA |
|  | GAGGAGAGCGGAGGCTTG |
| GSMUA_Achr11G10010_001 | CTCCAACACAGCACCCATC |
|  | GGACCCAGTCCAGCCATAG |
| GSMUA_Achr8G10740_001 | TGAACACGAAGGAGTTCCAA |
|  | ACTCCTCCTTTCTTATCTCTGACG |
| GSMUA_Achr9G05460_001 | CGAGGAGGAGGAAGAAGAGG |
|  | ATTGCTGTTAGCCAGCCAAG |
| GSMUA_Achr8G19810_001 | TCCTCTCCTGCCTCTGTCTC |
|  | GCCCATGAAGAGCAGGTC |
| GSMUA_Achr6G05710_001 | CCCGATGAGCACTTTGTGTA |
|  | CCACTGAGTCGAGCATGAAG |
| GSMUA_Achr7G25400_001 | TCATCAGAACCCTGCAACAG |
|  | AAGGAAGGAACGATGTCCTG |
| GSMUA_Achr4G15540_001 | CTGATCACCGAGGAGGAGTC |
|  | GAACAGAGAGTCGTCGTCACC |
| GSMUA_Achr10G23420_001 | CCATGGAGCACCTCATCAGT |
|  | TGAAATCCACCGTCAACGTC |
| GSMUA_Achr4G07230_001 | AGTAATTTGCCCTGTGGACTG |
|  | TCCTAGGCTCAGTCAATGTCC |
| GSMUA_Achr6G13630_001 | ACCGACGGACTGATGAACTC |
|  | GCATCACCAAGTGGGTCTTT |
| GSMUA_Achr8G14610_001 | GGTCGGATGCTCTTTTCGTA |
|  | AACATGTGTGACGGATCCAA |
| GSMUA_Achr2G15200_001 | CGTTCCCTGGTATGCCTGTA |
|  | TTATTGGCGCTGCTCTTGTT |
| GSMUA_Achr7G19340_001 | TGAGCACAACCATGGACATC |
|  | TTAGTTTTGAGCCGGTGGAT |
| GSMUA_Achr3G05670_001 | CTGATCTCACCGTCTCCAAGTT |
|  | CGATCTTGCCGTTGGAGAT |
| GSMUA_AchrUn_randomG17570_001 | CCAGATTCCGTCCACCTTT |
|  | GTTGGCAGTGTCGGATGAC |
| GSMUA_Achr4G03660_001 | AGACCCAAGACCGAGCTTTAG |
|  | TCATGCCACTGTTGCTTCTC |
| GSMUA_Achr11G17950_001 | CGCAATCTAAAGGAAGAGCCTA |
|  | CTGGCTTTGTTTCGGTAAGC |
| GSMUA_Achr6G05880_001 | GTCAAGGTTCTCAGGGTTGC |
|  | TTCTACTGGCAGTGCTCCTG |
| GSMUA_Achr8G01730_001 | GCAAGGATGAGAAGGACGAC |
|  | CTGCCTCGGCAAGTATATGG |
