# Supplementary material for: MusaWRKY71 Overexpression in Banana Plants Leads to Altered Abiotic and Biotic Stress Responses
Source: PLoS One. 2013 Oct 8;8(10):e75506. doi: 10.1371/journal.pone.0075506 (PMC3792942; doi:10.1371/journal.pone.0075506)
Supplement: Table S2 — PR protein genes and primers used in this study. (DOC) [file pone.0075506.s003.doc]

**Table S2** *PR-Protein* genes and primers

| **Gene Annotation** | **Primer Sequence 5’ to 3’** |
| --- | --- |
| GSMUA_Achr9T16540_001 | GAAGGCGACGGCTCTGTT |
|  | ACCGTCAGCACACATCCTC |
| GSMUA_AchrUn_randomT10460_001 | GCGGGATGGACTTCTACGA |
|  | CGTCGAAGGCGTAGCTGTA |
| GSMUA_Achr9T08440_001 | GCAGCACATGGTGAAGACA |
|  | GCGTGAGATGACTCCATCG |
| GSMUA_Achr6T17070_001 | CCTCCGAACTATGATGTCGTC |
|  | TCTGCAGGAAGGCGTTGTA |
| GSMUA_Achr3T08250_001 | TCCGTAAGGCAGCTCAACTT |
|  | CCGGGCAAGAACTTGTAGG |
| GSMUA_Achr3T08230_001 | AAGGAGCGGCTGGACTTT |
|  | ATGATTCCGGTCACCGTCT |
| GSMUA_Achr10T02850_001 | AGGGTGCTACACGGAGGTT |
|  | AGGTGCTTGTGCCATCATC |
| GSMUA_Achr6T31500_001 | ATGCCGCCAACTTCGAGAT |
|  | ACGTGGGGCTGAAGTCCATA |
| GSMUA_Achr10T11230_001 | GGAGCTCCTCCGGACTATG |
|  | CTCCTCCCATCTCCCGTAA |
| GSMUA_Achr7T21980_001 | ACGATCCTGCCGAAGAGAG |
|  | CGTCTCCTTCCTCGTCACC |
| GSMUA_Achr3T15660_001 | CTCACGTAGATGGCCGTTC |
|  | CTTGCCTTGTCCCCAGAAG |
| GSMUA_Achr2T08710_001 | TTCTCAGGGGCGTTACGG |
|  | TGGCAGAGGAGGAGTCGAA |
| GSMUA_Achr2T13210_001 | CACCACCTTAGCGCAAAAC |
|  | CGTCGATTCCGGTGTAGTC |
| GSMUA_Achr2T13220_001 | GGTCGGCAAATTACGCTTC |
|  | CGAAGAGGTTCTCCCCGTA |
| GSMUA_Achr5T27850_001 | ATCCGACAGCCGTCGTTC |
|  | TCCCAATCTCCTGGACGTG |
| GSMUA_Achr8T26540_001 | ACGCTGGTAAATCGTCTCAAA |
|  | GGAGAAGGAGGCGGTATCA |
| GSMUA_Achr4T23100_001 | AGGTCCTCAAACCCAGCTT |
|  | GCTGGTTGGCGTAATTCTG |
| GSMUA_Achr4T31970_001 | CAATGCTCCGACACCGTCT |
|  | AGCCGTAGGCTTGGCACT |
| GSMUA_Achr7T16510_001 | CTCCTCCCCCTGCTCTTC |
|  | TCGCCTGTCTGGCAGCTA |
| GSMUA_Achr2T13240_001 | GGTGTGTGCTATGGCCTTG |
|  | CCAGAAGATGTTCTCCCCATAA |
