# Supplementary material for: MusaWRKY71 Overexpression in Banana Plants Leads to Altered Abiotic and Biotic Stress Responses
Source: PLoS One. 2013 Oct 8;8(10):e75506. doi: 10.1371/journal.pone.0075506 (PMC3792942; doi:10.1371/journal.pone.0075506)
Supplement: Table S3 — Chitinase genes and primers used in this study. (DOC) [file pone.0075506.s004.doc]

**Table S3** Chitinase genes and primers

| **Gene Annotation** | **Primer Sequence 5’ to 3’** |
| --- | --- |
| GSMUA_Achr7T20770_001 | CAGCAAGACCTCCTGTGGAT |
|  | GCTCCAGGTATTCTGGATGG |
| GSMUA_Achr9T02370_001 | ATCGTGACGCAGAGCTTCTT |
|  | CATCGCAATAGTCCCTCGAT |
| GSMUA_Achr6T21330_001 | ATGAGACCGGACATTTCTGC |
|  | GCGGTCTTGAAGGAGATGAC |
| GSMUA_Achr8T24270_001 | ATGAGCCCAAGCCAAGACTA |
|  | AGGCTGCTTCTTCTTCATCG |
| GSMUA_Achr3T14710_001 | TCGTGCCATGAGGTCATTATC |
|  | TAAAGAGATCGCCGATAGCAGT |
| GSMUA_Achr6T36390_001 | GAAACTGGAAGCCCACAAAG |
|  | GATGGATTGAAAGGCACCTG |
| GSMUA_Achr3T06330_001 | CCACCAAGAACGACACATTG |
|  | GATGCGGTAGAAGGATCGAA |
| GSMUA_Achr3T26910_001 | CACGAAGAAGAGGGAGATCG |
|  | CGGCCCGTAGTTGTAGTTGT |
| GSMUA_Achr3T25100_001 | CTCGGTAAGGCTCTCCATTC |
|  | ATCCAGCTGAGAGGAGCATT |
| GSMUA_Achr5T19050_001 | ATGGGAGTTTGGGAGACAAC |
|  | GACCGTAGGTTCCGAAGTGT |
| GSMUA_Achr8T23500_001 | CACCTCGGTCTCTTCTTTCC |
|  | AGATCAACCTTCAGCCCATC |
| GSMUA_Achr8T16460_001 | GATCTGTGGGTGAGCTTTGA |
|  | CCTGTGGTGCTACAGAAGGA |
| GSMUA_Achr1T07320_001 | CCCGGCAAGAAGTACTATGG |
|  | CACTCCAACCCTCCGTTAAT |
| GSMUA_Achr6T30280_001 | AGACGTGGAAGCTCAAGGAC |
|  | CCGCTGGGCATAGTTTATCT |
| GSMUA_Achr8T27900_001 | TGGGACCAAGGTGTACCTCTC |
|  | CGTAGTTGGACGCAGTCTTGA |
| GSMUA_Achr8T27880_001 | GCGAAGCAGCTCTACGACTT |
|  | AGCACCTGGTTGGTGAGGT |
| GSMUA_Achr8T32980_001 | AGGTGTTCCTCGGACTCACT |
|  | GACGACGCCACCAAAGTAG |
| GSMUA_AchrUn_randomT11990_001 | AGCCAAGACTACTGCGATCC |
|  | ATCATGAGCTGAAGGCTGCT |
| GSMUA_Achr9T25580_001 | TATGGGCAGGTCATCGACTA |
|  | GAAGAGTCTGCAGCCCAGAT |
| GSMUA_Achr3T26900_001 | TCTGGATGACTCCTCAGTCG |
|  | TGGCTGTAGCAGTCCAAGTT |
| GSMUA_Achr9T16770_001 | TCAGATCTCCCATGAGACCA |
|  | CCACTGTTTCCGGATTCTTC |
| GSMUA_Achr7T23350_001 | CGGATACCAGGAGGCATTT |
|  | CGAGCAAGAAGGCTTGAGAT |
| GSMUA_Achr8T27910_001 | CTACGTGTGGGTGCAGTTCT |
|  | AACACTGCTCTTCACTTTCTCG |
| GSMUA_Achr3T26890_001 | TCTGGATGACTCCTCAGTCG |
|  | AATGTGGCTGTAGCTGCTGT |
| GSMUA_Achr3T22670_001 | AGCAACAGAGGCAAGAAGGT |
|  | GGCAGTACGTGCGATATGAG |
| GSMUA_AchrUn_randomT12350_001 | CTCATGCATTGGCGTCTACT |
|  | GCAGAACCACCCAAGAAACT |
