# Supplementary material for: MusaWRKY71 Overexpression in Banana Plants Leads to Altered Abiotic and Biotic Stress Responses
Source: PLoS One. 2013 Oct 8;8(10):e75506. doi: 10.1371/journal.pone.0075506 (PMC3792942; doi:10.1371/journal.pone.0075506)
Supplement: Table S4 — NPR1 genes and primers used in this study. (DOC) [file pone.0075506.s005.doc]

**Table S4** NPR1 genes and primers

| **Gene Annotation** | **Primer Sequence 5’ to 3’** |
| --- | --- |
| GSMUA_Achr3T25880_001 | CATTTCTTCCCACGGTGTTC |
|  | CAGCCTTGCTCCTAACTGCT |
| GSMUA_Achr7T09190_001 | GAACAGTGGAACTCGGGAAG |
|  | TACAATGCCGACCGATGTT |
| GSMUA_Achr6T00950_001 | GGCACGAATGAGAGCACTTT |
|  | ACAAGGCAGACCTGTCGAAT |
| GSMUA_Achr2T16670_001 | CTAGAACAGTGGAACTTGGGAAG |
|  | CCCTACAGACGTCGATGATG |
| GSMUA_Achr4T32060_001 | CGGCGATATTTTCCTCACTG |
|  | CTTGCAAACGTGTGATGCTT |
| GSMUA_Achr8T05490_001 | GCAACTTGGGATGCATTTCT |
|  | TAGAAGACGAAGCGGCAGAG |
| GSMUA_AchrUn_randomT01860_001 | CCAGGATGGAAGCTCTATCAA |
|  | GATGAAGCAGAGGCAGAACC |
| GSMUA_Achr5T16220_001 | TGACGTCCGACTTCCTCTTC |
|  | CCACCACTTGTGTGATTTGC |
| GSMUA_Achr5T02120_001 | CGCTATTTTCCCAACTGTTCA |
|  | AATCCTTAGGCAGCGATGAG |
